# Predictive value of serum inflammatory factors high-sensitivity C-reactive protein combined with high level of lipoprotein-associated phospholipase A2 for the onset of ischemic stroke

**DOI:** 10.5937/jomb0-56633

**Published:** 2025-10-28

**Authors:** Dajun Gu, Yaojin Zuo, Tao Zhang

**Affiliations:** 1 Chongqing Jiangjin District Traditional Chinese Medicine Hospital, Brain Disease Ward 1, Chongqing, 402260, China; 2 People's Hospital of Chongqing Liangjiang New Area, In-patient Dept of Neurology, Chongqing, 401120, China; 3 Chongqing Hospital of Jiangsu Province Hospital (The People's Hospital of Qijiang District Chongqing), In-patient Dept of Neurology, Chongqing, 401420, China

**Keywords:** IS, hs-CRP, Lp-PLA2, inflammation factors, predictive value, IS, hs-CRP, Lp-PLA2, inflamatorni faktori, prediktivna vrednost

## Abstract

**Background:**

The aim of this study was to carried out an exploration of the predictive value of serum high-sensitivity C-reactive protein (hs-CRP) plus high levels of lipoprotein-associated phospholipase A2 (Lp-PLA2) for the onset of ischemic stroke (IS). This study extends the understanding of their interplay by highlighting their mechanistic contributions to vascular inflammation and plaque instability, factors crucial in IS onset.

**Methods:**

526 IS patients were selected as the experimental group (EG). During the same period, 463 healthy individuals served as the control group (CG). The levels of Lp-PLA2, myeloperoxidase (MPO), total cholesterol (CHO), low-density lipoprotein cholesterol (LDL-C), triglyceride (TG), hs-CRP, and serum ferritin (SF) in the serum of subjects were compared. The predictive efficacy of combination of two for the onset of IS was assessed.

**Results:**

The levels of Lp-PLA2, MPO, CHO, LDL, TG, hs-CRP, and SF in patients with IS were all markedly higher as against the CG (P&lt;0.05). Multivariate Logistic regression analysis (MLRA) suggested that both hs-CRP and Lp-PLA2 were independently associated with the risk of IS (OR=1.334, 95% CI=1.713~1.954; 1.251, 1.011~1.921). The ROC curve analysis revealed that the predictive efficacy for IS of hs-CRP in combination with Lp-PLA2 (area under the ROC curve (AUC)=0.786) was markedly better as against hs-CRP alone (0.713) or Lp-PLA2 alone (0.698) (P&lt;0.05). Mechanistically, their interaction may exacerbate vascular inflammation, promoting plaque instability, a crucial process in IS development.

**Conclusions:**

This study reinforces that the combined detection of hs-CRP and Lp-PLA2 significantly improves IS risk prediction by offering a more comprehensive assessment of inflammatory and atherosclerotic status. Their interplay suggests potential therapeutic targets for preventing IS.

## Abstract (sr)

**Uvod:**

Cilj ove studije bio je istraživanje prediktivne vrednosti serumskog visokosenzitivnog C-reaktivnog proteina (hs-CRP) i visokih nivoa lipoproteinima povezanog fosfolipaze A2 (Lp-PLA2) za nastanak ishemijskog moždanog udara (IS). Ova studija proširuje razumevanje njihovog međusobnog delovanja, ističu i njihove mehanističke doprinose vaskularnoj inflamaciji i nestabilnosti plaka, faktore ključne za nastanak IS.

**Metode:**

526 pacijenata sa IS odabrano je kao eksperimentalna grupa (EG). Tokom istog perioda, 463 zdrave osobe služile su kao kontrolna grupa (CG). Upoređeni su nivoi Lp-PLA2, mieloperoksidaze (MPO), ukupnog holesterola (CHO), holesterola niskog gustinskog koeficijenta (LDL-C), triglicerida (TG), hs-CRP i serumskog feritina (SF) u serumu ispitanika. Procena prediktivne efikasnosti kombinacije ova dva faktora za nastanak IS je izvedena.

**Rezultati:**

Nivoi Lp-PLA2, MPO, CHO, LDL, TG, hs-CRP i SF kod pacijenata sa IS bili su značajno viši u poređenju sa CG (P&lt;0.05). Multivariatna logistička regresija (MLRA) je pokazala da su i hs-CRP i Lp-PLA2 nezavisno povezani sa rizikom od IS (OR=1.334, 95% CI=1.713č1.954; 1.251, 1.011č1.921). Analiza ROC krive je pokazala da je prediktivna efikasnost za IS kombinacije hs-CRP i Lp-PLA2 (površina ispod ROC krive (AUC)=0.786) bila značajno bolja u poređenju sa samim hs-CRP (0.713) ili samim Lp-PLA2 (0.698) (P&lt;0.05). Mehanistički, njihovo delovanje može pogoršati vaskularnu inflamaciju, podstičući i nestabilnost plaka, ključni proces u razvoju IS.

**Zaključak:**

Ova studija potvrđuje da kombinovano otkrivanje hs-CRP i Lp-PLA2 značajno poboljšava prediktivnu procenu rizika od IS, nudeći i sveobuhvatniju procenu inflamatornog i aterosklerotskog statusa. Njihovo međusobno delovanje sugeriše potencijalne terapijske ciljeve za prevenciju IS.

## Introduction

IS stands as a major contributor to mortality and chronic disability globally, representing a substantial burden to individuals, families, and society [1].[2] Despite certain advancements in the understanding of its pathophysiological mechanisms, early prevention and accurate prediction still faced great challenges. Inflammation is pivotal in the etiology and progression of IS, and the detection of inflammatory markers is of significant importance for the early identification of high-risk groups [3] [4].

hs-CRPis an acute-phase reactant protein synthesized by the liver, present in trace amounts in the body but markedly elevated by inflammatory responses. hs-CRP is widely recognized as an important marker of inflammation and cardiovascular diseases and is extensively used for risk assessment of cardiovascular diseases [5]. Increased hs-CRP levels obviously correlated with the development of IS [6]. High levels of hs-CRP are considered a marker of systemic inflammatory status, possibly reflecting the presence of atherosclerosis and the formation of unstable plaques [7] [8]. Some studies indicated that hs-CRP could not only serve as a predictive factor for IS but also be adopted for assessing the prognosis of stroke patients. The levels of hs-CRP are closely related to the severity of stroke patients’ conditions, functional recovery, and long-term prognosis [9]. The detection of hs-CRP is meaningful in clinical practice, especially in primary and secondary prevention. By detecting the levels of hs-CRP, doctors could identify high-risk patients earlier and take corresponding intervention measures [10].

Lp-PLA2is an enzyme closely related to atherosclerosis and vascular inflammation [11] [12], mainly secreted by macrophages, and its elevated levels are also considered an independent predictor of cardiovascular events, acting in atherosclerosis [13]. Lp-PLA2 intensifies arterial inflammation and plaque instability by hydrolyzing oxidized phospholipids, producing inflammatory and cytotoxic products (such as oxidized fatty acids) [14]. Although the independent roles of hs-CRP and Lp-PLA2 in predicting and assessing IS had been widely studied, there were relatively fewer studies on the combined application of the two to improve predictive efficacy.

This study hypothesizes that hs-CRP and Lp-PLA2 interact synergistically, enhancing predictive accuracy for IS by reflecting distinct yet complementary inflammatory pathways. By elucidating their mechanistic relationship, this study aims to provide novel insights into IS risk stratification and prevention.

## Materials and methods

### Subjects

This article retrospectively analyzed the data of 526 patients with IS treated at Chongqing JiangjinDistrict Traditional Chinese Medicine Hospital from January 2021 to December 2023 as the EG. Another 463 healthy individuals from the physical examination center of Chongqing Jiangjin District Traditional Chinese Medicine Hospital from June 2021 to November 2023 served as the CG. The EG had 261 men and 265 women, aged (57.86±9.61) years. The CG had 237 men and 226 women, aged (58.45±11.72) years; the contrast of gender, age, and body mass index among the subjects suggested no visibledistinction (P >0.05). All patients clearly understood the content of the trial, voluntarily signing the informed consent form, and this trial was also reviewed and obtained the approval by Chongqing Jiangjin District Traditional Chinese Medicine Hospital Ethics Committee.

Inclusion: (1) Patients with IS in the EG must meet the diagnostic criteria for IS published by the International Stroke Association (ISA) in 2021 and be confirmed by imaging (CT or MRI) (Bernhardts2023); (2) All subjects were aged 18 and above. (3) Patients with IS were admitted to the hospital for treatment within 72 hours of onset; (4) The vital signs of the patients were monitored and stable, and they had the tolerance for treatment; (5) The participants and their legal guardians provided consent for trial involvement and executed the informed consent documents.

Exclusion: (1) People with severe mental illness or intellectual disability, affecting their understanding and cooperation with intervention measures; (2) Those with severe heart, liver, kidney dysfunction, malignant tumors, severe infections, etc.; (3) People with autoimmune diseases: such as systemic lupus erythematosus, rheumatoid arthritis; (4) A history of acute or chronic inflammatory diseases within one month before the start of the trial; (5) Those who were taking medications affecting inflammation or lipid metabolism within one month before the start of the trial, such as statins, immunosuppressants; (6) Other important comorbidities or clinical conditions, such as severe anemia, malignant tumors, severe cardiovascular diseases, that might affect the interpretation of the study results.

### Sample collection

All subjects had 6 mL of fasting elbow venous blood collected the next morning after admission, in a resting state. First, anticoagulant treatment was performed, and the collected 6 mL blood sample was divided into two parts. 3 mL of blood was placed in an anticoagulant tube containing EDTA or heparin, gently mixed, and then centrifuged at 4°C at 3,000 rpm for 15 min. Later, the plasma was collected, storingat -80°C for later use to avoid repeated freezing and thawing, for the detection of MPO and Lp-PLA2 levels. Then, blood lipid detection was performed, and the remaining 3 mL of blood was placed in a regular biochemical tube for the detection of CHO, TG, high-density lipoprotein cholesterol (HDL-C), and LDL-C.

### Detection methods

ELISA was used to determine the serum Lp-PLA2, MPO levels.The reagents were provided by Tianjin Kangerke Biological Technology Co., Ltd.

The serum TG, CHO, and LDL were measured by the enzymatic method. The measurement instrument was the Hitachi 7600 automatic biochemical analyzer. The reagents were provided by Beijing Strong Biotechnologies, Inc.

The nephelometric turbidimetric method was employed for determining the serum hs-CRP and SF.

### Statistical processing

SPSS19.0 software was employed, and the measurement data were presented by mean ± d(x̄±s). The Logistic P regression model could be used to analyze the risk factors for IS, and the diagnostic test and ROC curve were used to determine the assessment threshold of the model. The Youden index in the diagnostic test was used to determine the optimal threshold, and the ROC curve was adopted to assess the diagnostic value of hs-CRP plus Lp-PLA2 and the use of hs-CRP or Lp-PLA2 alone for IS. The difference was statistically considerable with *P* <0.05.

## Results

### Contrast of test results in subjects

**Figure 1:**
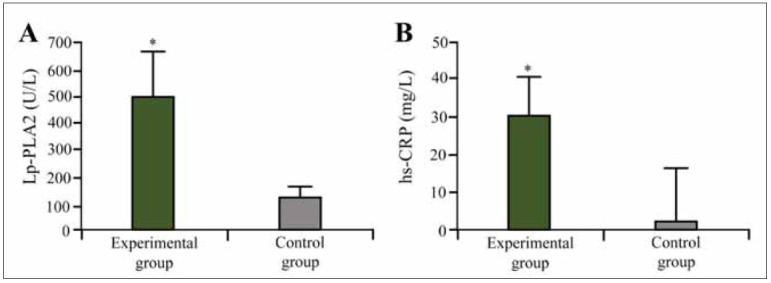
Lp-PLA2 and hs-CRP test results in subject.<br>(Note: A:Lp-PLA2; B: hs-CRP; * as against the CG, *P* <0.05)

In this study, the levels of Lp-PLA2 and hs-CRP were significantly elevated in IS patients compared to the control group (P<0.05). The mean Lp-PLA2 level in IS patients was (487.5±74.6) U/L, while in the control group, it was (121.4±31.3) U/L, demonstrating a marked increase (Figure 1A). Similarly, hs-CRP levels were significantly higher in IS patients, with a mean value of (30.4±7.62) mg/L compared to (2.53±0.32) mg/L in the control group. These findings indicate a strong association between inflammation markers and IS risk (Figure 1B).

Additionally, individual variations in Lp-PLA2 and hs-CRP levels were observed. The highest Lp-PLA2 level recorded in IS patients was 720 U/L, while the lowest was 350 U/L. Similarly, hs-CRP values ranged from 18.2 mg/L to 42.7 mg/L among IS patients, indicating substantial inflammatory variation within the group.

### Contrast of serum CHO, LDL, and TG test results in the subjects

**Figure 2:**
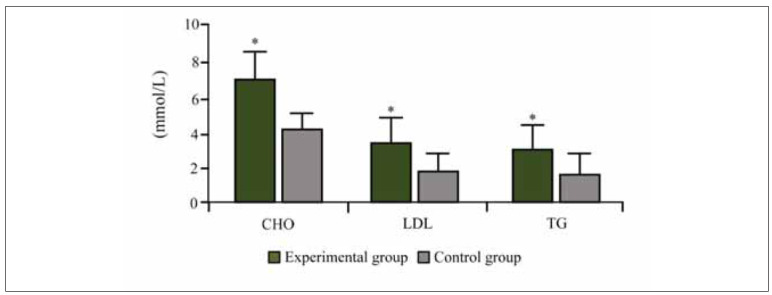
Contrast of CHO, LDL, and TG in subjects.<br>(Note: * as against the CG, P <0.05)

The serum CHO, LDL, and TG levels were also significantly higher in IS patients than in controls. The CHO level in IS patients was (7.13±1.06) mmol/L, whereas in the control group, it was (4.16±1.03) mmol/L. LDL levels followed a similar trend, being (3.43±0.75) mmol/L in IS patients and (1.93±0.47) mmol/L in the control group (P<0.05). TG levels were significantly elevated in IS patients at (2.75± 0.82) mmol/L versus (1.76±0.61) mmol/L in controls. These lipid profile differences suggest a direct relationship between lipid metabolism disturbances and IS risk (Figure 2).

Interestingly, patients with the highest LDL levels (>4.0 mmol/L) also exhibited significantly elevated hs-CRP levels (>35 mg/L), suggesting a possible correlation between lipid metabolism dysfunction and systemic inflammation in IS pathogenesis.

### Serum SF and MPO test results of the subjects

**Figure 3:**
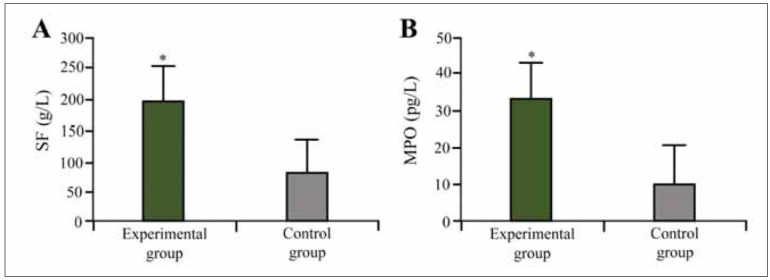
Serum SF and MPO test results in subjects.<br>(Note: A:SF; B:MPO; * as against the CG, *P* <0.05)

Interestingly, patients with the highest LDL levels (>4.0 mmol/L) also exhibited significantly elevated hs-CRP levels (>35 mg/L), suggesting a possible correlation between lipid metabolism dysfunction and systemic inflammation in IS pathogenesis (Figure 3A). Similarly, MPO levels were (32.5±8.29) pg/L in IS patients compared to (9.35±3.21) pg/L in controls (P<0.05). These findings support the hypothesis that increased inflammatory activity contributes to IS pathogenesis (Figure 3B).

Patients with the most severe IS cases (NIHSS >15) exhibited the highest MPO levels, averaging 38.2±9.1 pg/L, compared to 28.5±5.3 pg/L in patients with NIHSS scores below 10. This suggests that MPO could serve as a supplementary biomarker for stroke severity assessment.

### MLRA of IS

The results of Logistic regression model suggested that Lp-PLA2 and hs-CRP were risk factors for IS (*P*<0.05) (Table 1). Additional risk factors identified included CHO, TG, and MPO.

**Table 1:** MLRA of IS.

Variables	β	S.E	Wald	OR	95%CI	P
Lp-PLA2	0.135	0.081	6.613	1.251	1.011~1.921	0.004
hs-CRP	0.143	0.065	4.455	1.334	1.713~1.954	0.008
CHO	0.308	0.234	3.017	2.53	1.806~2.713	0.013
LDL	0.351	0.543	0.635	3.312	1.913~3.908	0.003
TG	0.513	0.654	1.654	1.375	1.146~2.937	0.004
SF	0.416	0.095	13.154	1.623	1.321~20.915	0.005
MPO	0.317	0.054	7.602	2.814	1.152~9.542	0.011

Clinically, an OR of 1.334 for hs-CRP suggests that for each unit increase in hs-CRP levels, the risk of IS increases by approximately 33.4%. Similarly, an OR of 1.251 for Lp-PLA2 implies a 25.1% higher likelihood of IS per unit increase. The strongest predictor among lipid markers was LDL (OR=3.312), suggesting a more than threefold increased risk of IS per unit increase. These findings reinforce the importance of monitoring these biomarkers in individuals at risk of IS, as elevated values significantly contribute to disease occurrence.

### AUC

**Figure 4:**
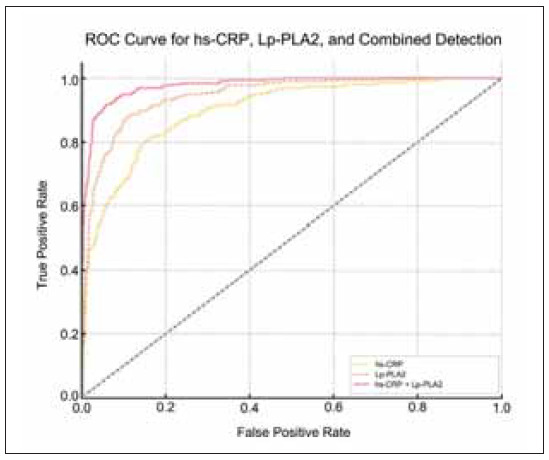
AUC.

In Figure 4 and Table 2, The ROC curve analysis demonstrated that the combined predictive efficacy of hs-CRP and Lp-PLA2 was superior to their individual predictive values. The AUC for hs-CRP alone was 0.713, while Lp-PLA2 alone had an AUC of 0.698. However, when both biomarkers were combined, the AUC increased to 0.786 (P<0.05), indicating enhanced predictive power.

**Table 2:** ROC curve analysis results of hs-CRP, Lp-PLA2, and their combined detection.

Variables	AUC	Sensitivity (%)	Specificity (%)	Optimal threshold
Lp-PLA2	0.698	72.5	71.4	4.5 mg/L
hs-CRP	0.713	68.3	78.9	190 ng/mL
Lp-PLA2+hs-CRP	0.786	83.6	83.5	_

The optimal thresholds for hs-CRP and Lp-PLA2 were determined using the Youden Index, which identifies the point that maximizes the sum of sensitivity and specificity, ensuring optimal classification performance. At the optimal threshold, the sensitivity and specificity of the combined detection were 83.6% and 83.5%, respectively, surpassing individual biomarker detection methods.

These values indicate a strong predictive capability compared to other models in the literature, where typical inflammatory biomarker models report sensitivity and specificity values around 70–80%. Previous studies using single inflammatory biomarkers for IS prediction reported AUC values around 0.72-0.75. In contrast, our combined detection method achieved an AUC of 0.786, suggesting that integrating hs-CRP and Lp-PLA2 enhances predictive accuracy beyond traditional models. The superior performance of this combined detection highlights its clinical applicability in IS risk assessment.

## Discussion

The important pathological basis of IS is atherosclerosis, and the essence of atherosclerosis is a chronic inflammatory response process [15] [16]. A single biomarker often has certain limitations when predicting disease risk. hs-CRP and Lp-PLA2 respectively reflect the state of inflammatory response and atherosclerosis, and their combined detection can provide more comprehensive pathophysiological information, thereby markedly improving the predictive accuracy of the risk of IS occurrence. hs-CRP is a sensitive marker of systemic inflammation, while Lp-PLA2 is closely correlated with the instability of atherosclerotic plaques (AP) [17]. The combined detection of the two can comprehensively assess the patient’s inflammatory state and the degree of atherosclerosis, providing clinicians with more detailed pathological information. This article found that hs-CRP and Lp-PLA2 have visible combined predictive value in predicting the risk of IS. These results provide new ideas and methods for the early prediction and prevention of IS.

IS is caused by the interruption of blood flow in the brain, leading to ischemia and necrosis of brain tissue. Numerous investigations have demonstrated that inflammation is crucial in the pathophysiology of IS [18]. Inflammatory factors are not only involved in the formation and progression of atherosclerosis but also affect the damage and repair of brain tissue after a stroke attack. Zheng et al. [19] indicated that inflammatory factors determine the prognosis of patients with ischemic stroke. Jianget al. [20] stated that inflammatory factors are closely related to the pathogenesis of IS. In this article, it was found that both hs-CRP and Lp-PLA2 were independent prediction factors of IS. hs-CRP is a classic inflammatory marker, and its elevated levels reflect a systemic inflammatory state. hs-CRP markedlycorrelates with the risk factors of cardiovascular diseases and stroke, and its mechanism may be related to the instability of AP [21]. Liu et al. [22] indicated that HS-CRP level has a regulating effect on the correlation between platelet count and clinical outcome in patients with ischemic stroke. C-reactive protein is a marker of inflammation, and Viktoria et al. [23] indicated that hs-CRP is somewhat associated with depressive symptoms in stroke patients.

Lp-PLA2 activity had an independent prognostic correlation with major coronary events. In addition, Lp-PLA2 is mainly combined with LDL, producing pro-inflammatory and cytotoxic products by hydrolyzing oxidized phospholipids, thereby exacerbating atherosclerosis and vascular inflammation [24] [25]. Specifically, Lp-PLA2 hydrolyzes oxidized phospholipids in LDL, generating lysophosphatidylcholine and oxidized fatty acids, which act as potent inflammatory mediators. These byproducts promote the recruitment and activation of macrophages, leading to further inflammation and the formation of foam cells within the atherosclerotic plaque. Moreover, Lp-PLA2-induced inflammation weakens the fibrous cap of the plaque, making it more prone to rupture and triggering the thrombotic events that lead to ischemic stroke. Increased levels of Lp-PLA2 were independently associated with the incidence of cognitive impairment in adults. Additionally, Xi et al. [26] remarked that increased Lp-PLA2 degrees link to a heightened risk of cardiovascular events or mortality in the Chinese middle-aged population. In this article, for each unit increase in hs-CRP, the likelihood of IS occurrence increased by about 27.8% (OR=1.278), and for each unit increase in Lp-PLA2, the likelihood increased by about 36.6% (OR=1.366). These results suggest that they can serve as independent prediction factors of IS, and combined detection can help enhance the assessment of the IS risk. The interplay between hs-CRP and Lp-PLA2 creates a vicious cycle of vascular inflammation and plaque vulnerability. Elevated hs-CRP levels contribute to endothelial dysfunction, promoting the adhesion and infiltration of inflammatory cells into the arterial wall. Lp-PLA2 exacerbates this process by generating pro-inflammatory mediators, amplifying the inflammatory cascade and increasing oxidative stress within the plaque. This chronic inflammatory milieu further destabilizes the plaque, increasing its susceptibility to rupture and subsequent ischemic stroke. Combined detection can improve the accuracy of stroke risk prediction, and the study results support these theories, indicating that both can serve as effective biomarkers for IS.

In this article, the ROC curve analysis suggested that the predictive performance of combined detection for the occurrence of IS was better than using either biomarker alone. The AUC value of the combined detection was markedly higher than the AUC values of the single method alone, indicating that combined detection has higher predictive accuracy. At the optimal threshold, the sensitivity and specificity of the combined detection were both better as against individual detections. This result emphasizes the effect of using hs-CRP and Lp-PLA2 together in the risk assessment of IS, which helps to improve the ability to identify high-risk patients early, thus achieving more effective prevention and intervention measures [17] The improved predictive accuracy offered by combined detection of hs-CRP and Lp-PLA2 provides clinicians with a valuable opportunity for early intervention. By identifying individuals at higher risk, lifestyle modifications such as diet and exercise, along with pharmacological interventions like statins or anti-inflammatory agents, can be implemented to mitigate the risk of IS. In addition to predicting the risk of IS, the combined detection can also be used to assess the long-term prognosis of patients. Elevated degrees of hs-CRP and Lp-PLA2 correlate with inferior functional rehabilitation and an escalated likelihood of recurrence, which helps doctors to manage and follow up patients more effectively during the treatment process [27]. The main findings of this article provide new ideas and methods for the early prediction and prevention of IS.

While this study focused on the combined predictive value of hs-CRP and Lp-PLA2 for IS risk, it is important to acknowledge that other inflammatory markers may also play a significant role in IS pathogenesis. For instance, studies have shown that elevated levels of fibrinogen, a key mediator of coagulation and inflammation, are associated with an increased risk of IS and may contribute to poor functional outcomes [28]. Moreover, recent research suggests that microRNAs, small non-coding RNA molecules that regulate gene expression, may also be involved in the inflammatory processes underlying IS [29] [30]. Further research is needed to explore the complex interplay of these and other inflammatory markers in IS development and progression.

This article also has some limitations. Although various potential confounding factors were included for adjustment, there may still be unidentified confounding factors affecting the results. Future studies can explore how to apply the detection results to individualized medical plans to optimize the prevention and treatment strategies for IS.

## Conclusion

This article demonstrated that the combination of hs-CRP and Lp-PLA2 has visible predictive value in predicting the risk of IS occurrence. The degrees of hs-CRP and Lp-PLA2 in patients with IS weremarkedly higher as against the healthy CG. MLRA and ROC curve analysis further confirmed that the combined detection can markedly improve the predictive efficacy for the risk of IS. Based on these findings, the combined detection can serve as an effective early warning tool for IS, aiding in the early identification of high-risk groups and timely intervention, thereby reducing the risk of IS occurrence. Future long-term follow-up studies can assess the impact of hs-CRP and Lp-PLA2 degrees on the longterm prognosis and risk of recurrence in patients with IS, providing more comprehensive guidance for clinical management.

## Dodatak

### Conflict of interest statement

All the authors declare that they have no conflict of interest in this work.
